# Inferior Left Atrial Diverticulum Communicating with the Right Atrium or Inferior Vena Cava: Prevalence and CT Features

**DOI:** 10.3390/jcdd13050215

**Published:** 2026-05-17

**Authors:** Hae Jin Kim, Sung Goo Park, Sung-A Chang, Jinyoung Song, Ji Hyuk Yang, Sung Mok Kim, Yeon Hyeon Choe

**Affiliations:** 1 Department of Radiology, Daejeon Eulji Medical Center, Eulji University, Daejeon 35233, Republic of Korea; haziness@eulji.ac.kr; 2 Department of Radiology, Samsung Medical Center, School of Medicine, Sungkyunkwan University, Seoul 06351, Republic of Korea; sunggoo.park@samsung.com (S.G.P.); sungmok_kim@hanmail.net (S.M.K.); 3 Cardiovascular Imaging Center, Heart Vascular Stroke Institute, Samsung Medical Center, School of Medicine, Sungkyunkwan University, Seoul 06351, Republic of Korea; sunga.chang@samsung.com; 4 Division of Cardiology, Department of Medicine, Heart Vascular Stroke Institute, Samsung Medical Center, School of Medicine, Sungkyunkwan University, Seoul 06351, Republic of Korea; 5 Department of Pediatrics, Samsung Medical Center, School of Medicine, Sungkyunkwan University, Seoul 06351, Republic of Korea; amyjys.song@samsung.com; 6 Division of Cardiovascular Surgery, Department of Cardiothoracic Surgery, Samsung Medical Center, School of Medicine, Sungkyunkwan University, Seoul 06351, Republic of Korea; jh1.yang@samsung.com

**Keywords:** left atrium, diverticulum, shunt, atrial septal defect, computed tomography

## Abstract

**Purpose:** To evaluate the prevalence and cardiac CT features of inferior left atrial diverticula (ILAD) communicating with the right atrium (RA) or inferior vena cava (IVC), a novel type of interatrial communication. **Materials and Methods:** This retrospective study included 11,512 consecutive patients who underwent cardiac CT. CT features and prevalence of ILAD communicating with the RA or IVC were analyzed. Shunts were defined as anatomical defects between the two structures with or without visible contrast flow. In a subset of the patients we compared interatrial septal aneurysm (*n* = 20) and ILAD without shunt (*n* = 66), assessing the involvement of a wedge-like fatty space bordered by both atria, IVC and coronary sinus. **Results:** There were 33 patients (19 males and 14 females; aged 59.8 ± 11.2 years; age range, 18–87 years) with ILAD with shunts (ILADSs). The prevalence of ILADSs was 4.2% (33/783) among ILAD and 0.3% (33/11,512) among all patients. Maximal dimensions of ILAD were 17.6 ± 9.9 mm (range, 5.3–41.0 mm). Mean ostial diameters of ILAD and mean sizes of shunts were 6.2 ± 5.6 mm and 3.2 ± 2.9 mm, respectively. Shunts were larger than 5 mm in 6 patients (15.2%) and larger than 3 mm in 10 patients (30.3%). In 30 patients who underwent transthoracic echocardiography, ILADSs were not identified at echocardiography. CT showed involvement of the wedge-like fatty space for all ILAD and for no cases with interatrial septal aneurysm. **Conclusions:** Cardiac CT enables detection of incidental ILADSs unrecognized at echocardiography.

## 1. Introduction

Left atrial diverticula (LAD) are cavities of various shapes and dimensions that are visualized as pouch-like outgrowths from the atrial wall [1,2,3]. It can occur in various regions of the left atrium (LA), most frequently along the superior walls [4,5]. They are generally asymptomatic but have recently been allegedly associated with atrial arrhythmias and thromboembolism in some case reports [6,7]. Radiofrequency energy delivery in LAD can cause trap and wall perforation [6,8]. It has been suggested to carefully search for atrial diverticula before ablation procedures and to remove atrial diverticula to eliminate the potential source of emboli despite anticoagulation [9,10].

LAD arising from the inferior wall of the LA are relatively uncommon and less well characterized in the literature [11]. These inferiorly located diverticula are typically situated adjacent to the right atrium (RA) and the inferior vena cava (IVC), and a subset of such diverticula shows direct connections with the RA, the IVC, or even both, distinct from classical atrial septal defects (ASDs) or patent foramen ovale (PFO). The discovery of these structures is new and their clinical significance remains unclear. In patients with larger communications, their clinical findings should be similar to those of conventional interatrial septal defects. However, detection of the anomaly may not be easy in echocardiographic examinations because of their atypical locations. In cases of small communications with dimensions similar to those of PFO, their clinical consequences are difficult to predict. A clearer knowledge of these structures may inform management strategies.

Thus, the aim of this study was to investigate the prevalence and morphological characteristics of inferior LAD (ILAD) communicating with the RA, IVC, or both structures in patients who underwent electrocardiography (ECG)-gated cardiac computed tomography (CT).

## 2. Materials and Methods

### 2.1. Study Population

**Figure 1:**
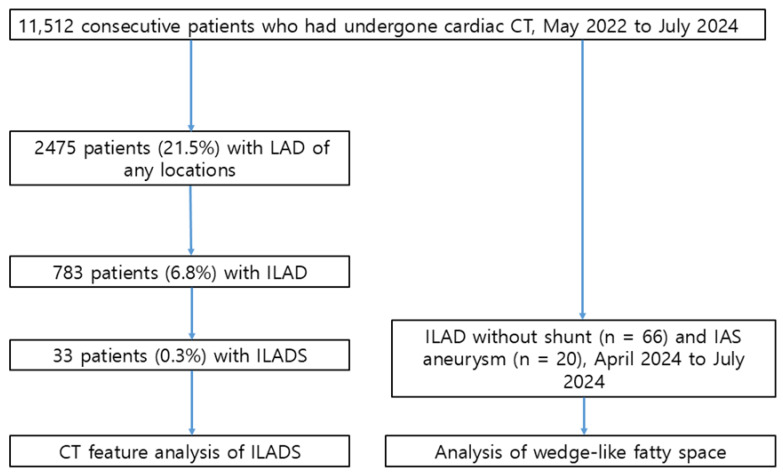
Patient flow diagram. Abbreviations: IAS, interatrial septal aneurysm; ILAD, inferior left atrial diverticula; ILADS, left atrial diverticula with shunts; LAD, left atrial diverticula.

We retrospectively reviewed the radiological reports of cardiac CT in 11,512 consecutive patients who had undergone cardiac CT between May 2022 and July 2024, including patients with ILAD with shunt (ILADS) (Figure 1). The Institutional Review Board (IRB) of Samsung Medical Center approved the study protocol and granted a waiver of informed consent due to its retrospective design. All patients belonged to the Asian race. The main indications for imaging included unexplained chest pain, angina pectoris, preoperative evaluation for noncoronary surgery, screening for coronary artery disease, planning of radiofrequency ablation in patients with atrial fibrillation, and determination of the patency of bypass grafts or stents.

There were 33 patients (19 males and 14 females; aged 59.8 ± 11.2 years; age range, 18–87 years) with ILADS. Patients had angina in seven (21.2%), dyspnea in one (3.0%) and no symptoms in the others (75.8%) (Table 1). Clinical diagnoses prior to cardiac CT examinations were atrial septal defect in nine (27.3%), atrial fibrillation in seven (21.2%), coronary artery disease in three (9.1%), Marfan syndrome in three (9.1%), aortic aneurysm in two (6.1%), and aortic valve stenosis in two (6.1%). Other clinical diagnoses included stroke, Takayasu’s arteritis, pericarditis, fibromuscular dysplasia, infective endocarditis, heart failure, mitral valve prolapse, pulmonary arteriovenous fistula, pulmonary artery thromboembolism, bicuspid aortic valve, ventricular septal defect, double-outlet right ventricle, and tetralogy of Fallot in one patient, respectively.

The locations, maximal sizes, types, and ostial diameters of ILAD, numbers of shunts, and sizes of shunts were analyzed. We reviewed echocardiography results, surgical records, and comorbidities.

Although ILAD are unique in appearance, interatrial septal aneurysm (IASA) or redundant interatrial septum may mimic ILAD. As ILAD are located in the triangular-shaped fatty space wedged by inferior walls of LA and RA on cardiac-short-axial CT images and bordered by suprahepatic IVC, LA and coronary sinus on transverse CT images, we compared ILAD and IASA or redundant interatrial septum on CT images. To compare findings in the triangular wedge-like fatty space, we included 20 consecutive patients (11 males and 9 females; aged 59.9 ± 16.7 years; age range, 24–86 years) with IAS and 66 consecutive patients (34 males, 32 females; 60.1 ± 16.0 years; age range, 17–83 years) with ILAD without shunts among those who had undergone coronary or cardiac CT during the period of April 2024 to July 2024.

### 2.2. Acquisition of CT Data

CT scans were performed using a second-generation or third-generation DSCT system (SOMATOM Definition Flash or Force; Siemens Healthineers, Forchheim, Germany). Metoprolol 50 mg was administered 1 h before the examination if the patient’s heart rate was >70/min. Nitroglycerin 0.4 mg was administered sublingually one minute before scanning. The CT acquisition delay time was calculated as the time of peak contrast medium attenuation in a region of interest in the ascending aorta plus 11 s after injection of 50–70 mL of nonionic contrast material (iomeprol 400 mgI/mL) at an injection rate of 4.0–4.5 mL/s and saline flush (30 mL, 4.0 mL/s). Acquisition parameters were 2 × 64 × 0.6 mm detector collimation resulting in 2 × 128 × 0.6 mm or 2 × 192 × 0.6 mm sections, 250 or 280 ms gantry rotation time, and 100 kV tube voltage. A real-time tube current modulation was performed with 320 reference mAs according to the precise shape of the patient’s body (CAREDose4D; Siemens Healthineers).

### 2.3. Echocardiography

Comprehensive two-dimensional and Doppler echocardiographic examinations were performed in a standard manner using commercially available echocardiographic devices (Vivid 7 or E9, GE Medical Systems, Milwaukee, WI, USA) [12,13]. All the echocardiographic recordings were interpreted by experienced cardiologists. Transthoracic echocardiography was performed in 30 patients (90.9%) and transesophageal echocardiography in seven patients.

### 2.4. Data Analysis

Transverse, cardiac short-axis, 4-chamber view, 2-chamber view, coronal, and sagittal reformatted CT images were used for detection of ILAD. Multiplanar reformatted images were used to analyze and measure ILADS using commercial software (Aquaris Intuition ver 4.4.13., TeraRecon, Durham, NC, USA). ILADSs were classified into morphological types including tubular, saccular, network-like or mixed pattern. Communication between the diverticula and the RA or IVC was defined as anatomical defects between the two structures with or without visible contrast flow or attenuation step up. The presence or absence of the communication or shunt was determined by two radiologists (YHC and HJK) in consensus. The size of the shunts were measured by the two observers twice at one-month intervals to calculate inter- and intraobserver variabilities. Continuous data are expressed as mean ± SD. Categorical variables are presented as percentages.

## 3. Results

Among the 11,512 patients, 2475 patients (21.5%) showed LAD of any location and 783 patients had ILAD (6.8%). Thirty-three (0.3%) patients had ILAD with a connection between LAD and RA, IVC, or both (Figure 1).

In patients with ILADS, tubular-type diverticula were found in 13 patients (39.4%), saccular-type in 10 (30.3%), tubulosaccular-type in 8 (24.2%) and network-type in 2 (6.1%). Maximal dimension or length of ILADS was 17.6 ± 9.9 mm (range, 5.3–41.0 mm) and larger than 5 mm in 19 patients (57.6%). In cases with saccular aneurysms, ILADS measured 9.5 ± 8.3 mm (range, 3.0–32.1 mm). The mean os diameter of ILADS was 6.2 ± 5.6 mm (range 1.7–22.9 mm), exceeding 5 mm in 15 patients (45.4%). Twelve patients (36.4%) showed two or more shunts from ILADSs. Average number of shunts was 1.6 (range, 1–4). Mean shunt size was 3.2 ± 2.9 mm (range, 1.0–13.4 mm). Shunt was larger than 5 mm in 6 patients (15.2%) and larger than 3 mm in 10 patients (30.3%). Draining sites of the shunts were RA in 16 (48.4%), IVC in 13 (39.4%), both RA and IVC in 3 (9.1%) and coronary sinus in 1 (3.0%). Associated LAD were found in the upper LA wall in 10 patients (30.3%), interatrial septum in 10 patients (30.3%), and lateral wall in 4 patients (12.1%).

CT revealed PFO in seven patients (21.2%) and sinus venosus-type separate ASD in two patients (6.1%).

Of the 30 patients who received transthoracic or transesophageal echocardiography, ILAD was not detected on echocardiographic assessment. Echocardiography identified shunts in nine patients (27.3%). In four cases, ILADS was incorrectly diagnosed as a secundum ASD. Additionally, in two patients with ASDs, ILADS was mistaken for another ASD. In seven patients with larger shunts (≥3 mm) into RA, echocardiography showed them in six patients (85.7%). In all 15 smaller shunts (<3 mm) into RA, they were not detected at echocardiography. On the contrary, larger shunts (≥3 mm) into IVC were detected in two (50%) of four patients. One (20%) of five smaller shunts (<3 mm) into IVC was detected at echocardiography.

**Figure 2:**
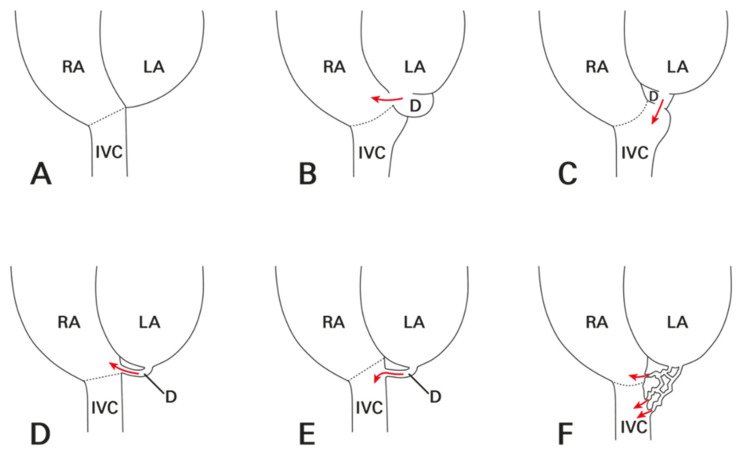
Representative diagram of normal atrial anatomy and left atrial diverticula with shunts. (**A**) Normal atria without diverticulum. (**B**) Saccular-type diverticulum communicating with RA. (**C**) Saccular-type diverticulum communicating with IVC. (**D**) Tubular-type diverticulum communicating with RA. (**E**) Tubular-type diverticulum communicating with IVC. (**F**) Network-like-type diverticulum communicating with RA and IVC. D represents diverticulum and arrows indicate direction of shunts. Dotted lines delineate valves of IVC. Abbreviations: IVC, inferior vena cava; LA, left atrium; RA, right atrium.

**Figure 3:**
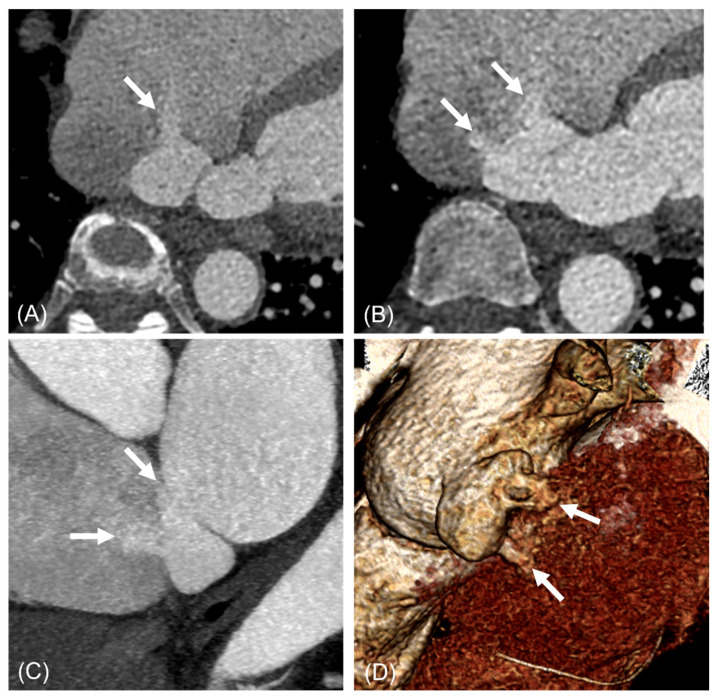
A 67-year-old woman with saccular type ILAD with multiple shunts into RA and IVC. Three jets of shunt flow heading toward RA (**A**–**C**) and one jet toward IVC (**C**) are visible (arrows). The ostial diameters of shunts were 3.1 mm, 9.1 mm, and 4.1 mm, respectively. Volume-rendered image shows saccular-type diverticulum and shunts ((**D**), arrows). Abbreviations: ILAD, inferior left atrial diverticulum; IVC, inferior vena cava; RA, right atrium.

**Figure 4:**
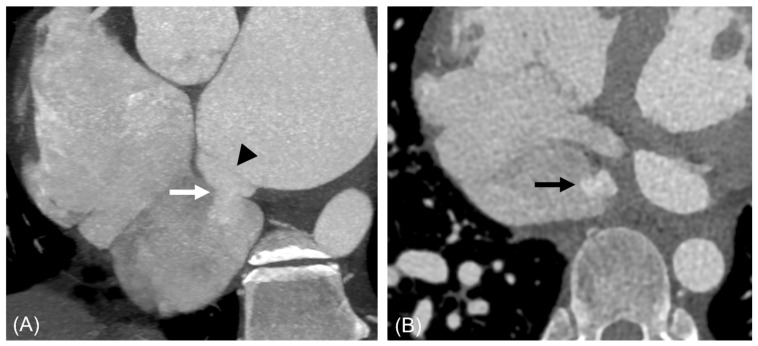
A 42-year-old man with saccular type ILAD communicating with IVC. Short-axis image (**A**) shows saccular diverticulum demarcated from LA by a membranous structure (arrowhead) and shunt (white and black arrows) below IVC valve (**A**,**B**). The shunt size measured 8 mm and was not visualized on transthoracic echocardiography. Abbreviations: ILAD, inferior left atrial diverticulum; IVC, inferior vena cava, LA, left atrium.

**Figure 5:**
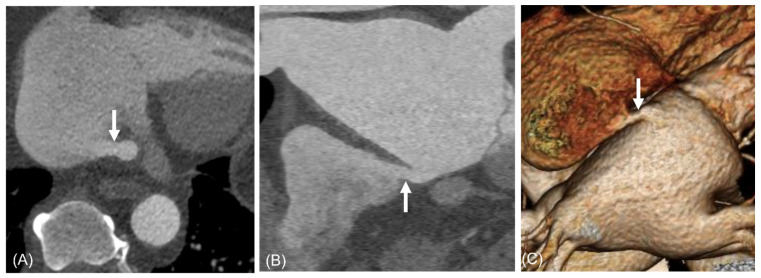
A 61-year-old man with tubular-type ILAD communicating with RA. Transverse (**A**), two-chamber view (**B**), and volume-rendered image (**C**) show tubular-type diverticulum with shunt (arrows) between both atria. Abbreviations: ILAD, inferior left atrial diverticulum; RA, right atrium.

**Figure 6:**
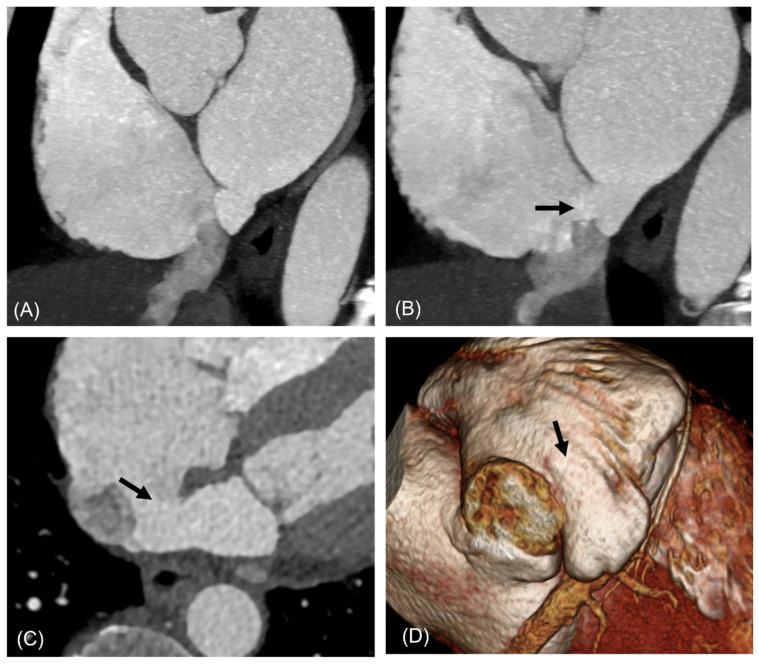
A 76-year-old woman with saccular-type ILAD communicated with RA. Short-axis view of cardiac CT scan shows saccular-type diverticulum originating from the inferior wall of LA (**A**) communicated with RA (**B**,**C**) (arrow). Three-dimensional reconstruction image shows saccular-type diverticulum (**D**), (arrow). The diverticulum was initially mistaken for an ASD on transthoracic echocardiography, because of its relatively large size and defect. Abbreviations: ASD, atrial septal defect; ILAD, inferior left atrial diverticula; LA, left atrium; RA, right atrium.

**Figure 7:**
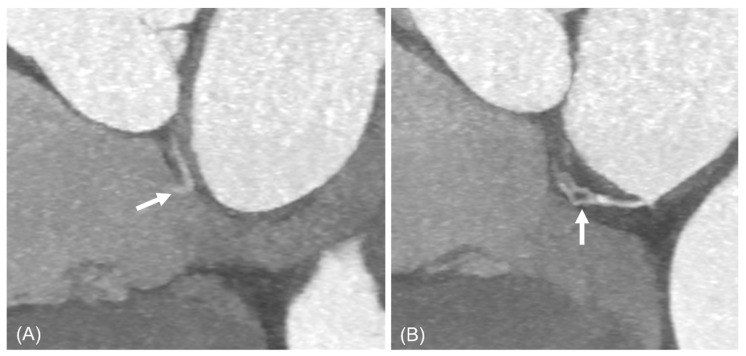
An 87-year-old woman with network-like-type diverticulum communicating with RA. The lesion was identified during the preoperative work-up for aortic arch aneurysm surgery. Short-axis images of cardiac CT scan show a shunt (**A**) (arrow) and spiderweb-shaped channels (**B**) (arrow) from inferior LA toward RA. Abbreviations: LA, left atrium; RA, right atrium.

Table 1 summarizes the clinical characteristics of the study population. Table 2 details the aneurysm location and draining site, with a corresponding diagram in Figure 2. CT images illustrating the morphological types of ILADS are presented in Figure 3, Figure 4, Figure 5, Figure 6 and Figure 7.

Surgery was performed to repair presumed ASD in two patients. In a 64-year-old female with multiple shunts, ILADSs were regarded as multiple ASDs. In another case of a 39-year-old male, a small tubular diverticulum with shunt was found as an unusual pouch-like structure located between IVC and coronary sinus.

**Figure 8:**
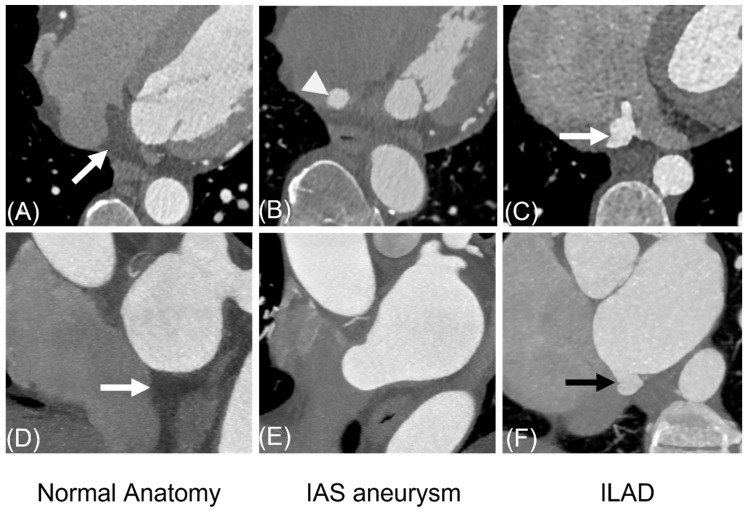
Comparison among normal anatomy, interatrial septal aneurysm (IASA), and ILAD on transverse (**upper panel**) and short-axial (**lower panel**) CT images. The wedge-like fatty space (white arrows in (**A**,**D**) remains intact in healthy individuals and those with IAS (**B**,**E**), where the aneurysmal area (arrowhead in (**B**)) is restricted to the right atrium. In contrast, ILAD (white and black arrows) (**C**,**F**) fills this fatty space. Abbreviations: IASA, interatrial septal aneurysm; ILAD, inferior left atrial diverticulum.

In a subset of patients comparing ILAD without shunt (*n* = 66) and IASA (*n* = 20), saccular-type ILAD were found in 44 patients (66.7%), tubular-type in 20 patients (30.3%) and network-type in 2 (3.0%). Maximal dimensions of ILAD were 12.4 ± 3.9 mm (range, 4.9–22.4 mm). Transverse CT and short-axial images showed a bulge into the wedge-like fatty space in 100% (66/66) (Figure 8). Stroke was associated with ILAD in 4.5% (3/66). In patients with IASA, maximal depth of bulge into the RA on four-chamber view or transverse CT images was 14.5 ± 3.5 mm (range, 8.0–19.4 mm). Transverse, four-chamber view and short-axial CT images showed no bulge into the triangular fatty space. Stroke was associated with an IASA in 5.0% (1/20).

Interobserver and intraobserver variabilities for calculating shunt sizes of ILAD showed high agreement (intraclass correlation coefficient: 0.95 for interobserver variabilities and 0.96 for intraobserver variabilities for two observers, respectively).

## 4. Discussion

This study systematically characterized ILAD that communicate with the RA or IVC, a novel type of interatrial communication not previously described in detail in the literature. Our study showed that the prevalence of ILADS was 0.3% among all patients. Shapes of ILADS were tubular (*n* = 13, 39.4%), saccular (*n* = 10, 30.3%), tubulosaccular (*n* = 8, 24.2%), and network-like appearance (*n* = 2, 6.1%). Maximal dimensions of ILAD were 17.6 ± 9.9 mm (range, 5.3–41.0 mm). Mean shunt size was 3.2 ± 2.9 mm. Shunts were larger than 5 mm in six patients (15.2%). In 30 patients evaluated by transthoracic echocardiography, ILD was not detected. In ILAD patients, lesions affected the wedge-like fatty space, but this did not occur with aneurysmal interatrial septum.

LAD is a common CT finding [14,15,16,17,18]. Genç et al. reported the frequency of LAD in 46.7% of 1305 patients [19]. In the study of Incedayi et al., LAD in all locations were found in 41% of 454 patients on CT [15]. Inferomedial diverticula were rare (0.7%) in their series. The size of LAD ranged 2 mm to 16 mm. Patel et al. found that 35% of 46 patients had LAD and accessory auricle, while De Ponti et al. observed a prevalence of 27.3% among 212 patients who underwent radiofrequency catheter ablation (RFCA) [6,16]. No literature has reported LAD connecting to RA or IVC via a shunt. In our cases, these shunt lesions were either absent or misidentified as ASD on echocardiography.

From a multimodality imaging perspective, cardiac CT and echocardiography should be regarded as complementary rather than competing techniques in the evaluation of ILADS. A structured imaging approach may help reduce diagnostic uncertainty and guide subsequent management decisions [20].

Mean shunt size (3.2 mm) of ILADS is similar to the dimension of PFO detected with cardiac CT in the literature [21,22]. Saremi et al. reported that middle tunnel diameter of a PFO shunt measured 2.5 ± 0.6 mm (range, 1.2–3.8 mm) [21]. In 15.2% of patients diagnosed with ILADS, the shunt size exceeded 5 mm. This finding suggests that the morphological characteristics of ILADS may have pathophysiological relevance in causing stroke. Larger or multiple communications can enable paradoxical embolization or cause cryptogenic stroke like PFO, while aneurysmal lesions provide sites for thrombus formation. However, at present, there are insufficient data to support an association between ILADS and increased cardioembolic risk or intervention.

Additionally, the ostial locations of ILADS differed from those observed in classical ASDs, which may contribute to challenges in visualizing them using transthoracic or transesophageal echocardiography. From a clinical perspective, it is important for radiologists and clinicians to consider this entity to prevent misclassification of interatrial shunts. Unrecognized ILADSs may result in an underestimation of a patient’s risk profile for paradoxical embolism or omission of this lesion during surgical or interventional procedures. Cardiac CT can assist in diagnosing this lesion, which might not be detected through echocardiography.

The ILADSs traverse different pathways through the fatty interatrial septum or the wedge-shaped fatty space located between the lower portions of both atria. The wedge-shaped fatty space serves as an anatomical marker that helps distinguish ILADS from aneurysmal interatrial septum, which can resemble ILAD. ILADSs are also distinguished from other types of interatrial shunts due to the involvement of the wedge-like fatty space. However, the wedge-like fatty space may not be present due to anatomical variations, left atrial enlargement, ASDs in inferoposterior septum or dilated coronary sinus.

Unroofed coronary sinus (UCS) is a rare congenital cardiac anomaly with complete or partial defects in the roof of the coronary sinus [23]. ILDASs are different from UCS, as there is direct communication between the LA and coronary sinus in patients with UCS, although a tubular form of UCS exists [24].

Limitations of this study include it being a retrospective single-center design with inherent selection bias. Second, the small sample size of patients with ILADSs restricts the capacity to assess clinical outcomes like stroke, arrhythmia, or pulmonary hypertension.

## 5. Conclusions

ILAD communication with the RA or IVC is a newly identified interatrial shunt detectable by cardiac CT, often overlooked in echocardiography. ILADS should be recognized as an uncommon but clinically relevant CT finding. 

## Figures and Tables

**Table 1:** Characteristics of patients with inferior left atrial diverticula with shunt.

Variable	ILADS (*n* = 33)
Age, years (mean ± SD, range)	59.8 ± 11.2 (18–87)
Male sex, *n* (%)	19 (57.6%)
Symptoms, *n* (%)	Asymptomatic, 25 (75.8%)
	Angina, 7 (21.2%)
	Dyspnea, 1 (3.0%)
Associated diseases *, *n* (%)	Atrial fibrillation, 7 (21.2%)
	Coronary artery disease, 3 (9.1%)
	Marfan syndrome, 3 (9.1%)
	Aortic valve stenosis, 2 (6.1%)
	Aortic aneurysm, 2 (6.1%)
	Atrial septal defect †, 2 (6.1%)
	None, 7 (21.2%)
	Others, 12 (36.4%)
Stroke history, *n* (%)	1 (3.0%)

* Clinical diagnoses overlap in five patients. † Atrial septal defects diagnosed with cardiac CT. Abbreviations: ILADS, inferior left atrial diverticula with shunt.

**Table 2:** The type, size, and draining site of ILADS.

Variables	Numbers
Type	
Tubular, *n* (%)	13 (39.4%)
Saccular, *n* (%)	10 (30.3%)
Tubulosaccular, *n* (%)	8 (24.2%)
Network-like appearance, *n* (%)	2 (6.1%)
Size	
Length of ILADS	17.6 ± 9.9 mm (range, 5.3–41.0 mm)
Os diameter of ILADS	6.2 ± 5.6 mm (range, 1.7–22.9 mm)
Average number of shunts	1.6 (range, 1–4)
Mean shunt size	3.2 ± 2.9 mm (range, 1.0–13.4 mm)
Draining site	
RA	16 (48.4%)
IVC	13 (39.4%)
RA and IVC	3 (9.1%)
Coronary sinus	1 (3.0%)

Abbreviations: ILADS, inferior left atrial diverticula with shunt; IVC, inferior vena cava; RA, right atrium.

## Data Availability

The original contributions presented in this study are included in the article. Further inquiries can be directed to the corresponding author.

## Abbreviations

The following abbreviations are used in this manuscript:

ASDs	Atrial septal defects
ILAD	Inferior left atrial diverticula
ILADS	Inferior left atrial diverticula with shunt
IVC	Inferior vena cava
LA	Left atrium
LAD	Left atrial diverticula
PFO	Patent foramen ovale
RA	Right atrium
